# Between access uncertainty and procedural immediacy: Users’ health-seeking determinants in a Brazilian Family Health Unit

**DOI:** 10.1016/j.aprim.2026.103561

**Published:** 2026-07-02

**Authors:** Daliane Soares Dantas Monteiro, Madson Caio dos Santos Dantas, Daira Soares Dantas Monteiro, Luiz Paulo Gomes dos Santos Rosa

**Affiliations:** a Medical Residency Program in Psychiatry, Brotherhood of the Holy House of Mercy of São Paulo (ISCMSP), São Paulo, Brazil; b Family and Community Medicine, Multicampi School of Medical Sciences, Federal University of Rio Grande do Norte (UFRN), Caicó, Brazil; c Federal University of Rio Grande do Norte (UFRN), Natal, Brazil; d Private University of the East (UPE), Presidente Franco, Paraguay; e Multicampi School of Medical Sciences, Federal University of Rio Grande do Norte (UFRN), Caicó, Brazil

Utilisation of emergency services for conditions considered primary care-sensitive generates system-wide overcrowding and avoidable costs.^1^ Brazil's Family Health Strategy (FHS), covering over 130 million people, organises primary health care (PHC) through multidisciplinary Family Health Units (FHUs).^2^ Despite the nationwide implementation of the FHS, this pattern persists. This study aimed to explore the perceptions of registered FHU users regarding their local health care network and the determinants of their choice between PHC and the local emergency service.

A qualitative study using focus groups was conducted at an FHU serving a socioeconomically vulnerable population in a medium-sized municipality in the Seridó region of Rio Grande do Norte, northeastern Brazil, in July 2025. Three focus groups (*n* = 21; 16 women, 5 men; age range 24–69 years, mean 50 years) were conducted – in ∼50 min, each – with purposively recruited frequent users from the seven distinct catchment micro-areas, a strategy that prioritises experiential density but underrepresents non-attenders and direct emergency department users. Data were analysed using Bardin's content analysis with inductive category derivation conducted primarily by the lead researcher,^3^ with iterative discussion among all co-authors; reporting followed the Consolidated Criteria for Reporting Qualitative Research (COREQ).^4^ The study was approved by a Research Ethics Committee (CAAE: 88091425.2.0000.5568). All participants provided written informed consent.

Content analysis yielded 77 coded units grouped into 20 subcategories and five overarching categories (Table 1). *Experience-based attribution of local health service roles* (*n* = 25) encompassed both users’ attribution of PHC-sensitive conditions to hospital care (“*Symptoms like vomiting, diarrhoea, high fever… I don’t think the health unit can handle it; you have to go to the hospital”*) and experiences of inter-service referral as system disorganisation, a pattern grounded in accumulated experiential knowledge of local service capabilities rather than familiarity with PHC's designated clinical scope.

**Table 1:** Content analysis categories and distribution of analytical units.

Category	Units of analysis (*n*)
Experience-based attribution of local health service roles	25
Unmet need for health system navigation support	8
Access-driven service seeking	24
Relational trust in the primary care team	9
Procedural immediacy as a marker of care effectiveness	11
Total	77

PHC = primary health care.

Participants’ accounts suggested that emergency service preference reflected coherent adaptive responses to PHC access unpredictability, shaped by direct experience of local service constraints. Guaranteed attendance and rapid intervention were perceived as markers of service resolution capacity.

Access constraints (appointment unpredictability, scheduling barriers, and guaranteed attendance at emergency departments) emerged as structural determinants diverting users towards emergency settings (“*Sometimes it's about access: I came, couldn’t get a slot, so I went to the hospital, knowing I’d be seen no matter what”*). Within Levesque et al.’s access framework,^5^ these findings reflect failures in the ‘availability and accommodation’ dimension, whereby organisational barriers reduce timely access to primary care and reinforce emergency departments as perceived guaranteed entry points.

*Relational trust* emerged as a partial counterweight to access-driven diversion, consistent with Starfield's PHC continuity attribute.^6^ *Procedural immediacy as a marker of care effectiveness* was the most analytically consequential. Users equated effective care with rapid procedures rather than with clinical problem resolution (“*I’d rather have the tooth pulled than come back to the unit more than once”*). Reinterpreted as rational adaptive choices under structural access constraints, these preferences suggest a practical reasoning within perceived structural limitations rather than clinical misjudgement: a pattern consistent with comparable findings in low- and middle-income country PHC settings.

Users’ health-seeking decisions therefore reflected coherent adaptive responses to perceived access uncertainty. Informational interventions alone would be insufficient without parallel reorganisation of PHC access conditions. Practical recommendations may include: ensuring reliable PHC appointment capacity; strengthening community health agents as navigational mediators; and implementing participatory communication strategies within PHC settings. These dimensions appear most effective when addressed simultaneously. Interpreting users’ behaviour as structural adaptation rather than a health system knowledge deficit may offer an actionable basis for PHC intervention design in similar contexts, directing attention towards access reorganisation as the primary target.

## Author contributions

**Daliane Soares Dantas Monteiro:** Conception and design; data acquisition; qualitative analysis; manuscript drafting; final approval.

**Madson Caio dos Santos Dantas:** Critical intellectual revision; editorial adaptation and internationalisation; final approval.

**Daira Soares Dantas Monteiro:** Support in data interpretation; critical revision; final approval.

**Luiz Paulo Gomes dos Santos Rosa:** Supervision; critical intellectual revision; final approval.

## Ethical considerations

Approved by an Institutional Review Board (CAAE: 88091425.2.0000.5568) in compliance with the Declaration of Helsinki and Brazilian National Health Council Resolutions 466/2012 and 510/2016. All participants provided written informed consent.

## Use of artificial intelligence

No generative AI tools were used in the preparation, writing, translation, analysis, or editing of this manuscript.

## Funding

No specific grant from funding agencies in the public, commercial, or not-for-profit sectors.

## Conflicts of interest

The authors declare no conflicts of interest.
